# 

**Published:** 1881-02

**Authors:** 


					To Readers and Correspondents.
Communications intended for publication, and exchange journals and pa-
pers, should be addressed to the Editor of the American Journal of Den-
tal Science, 259 N. Eutaw St., Baltimore, Md. All letters relating to the
business of the journal, or containing cash remittances, to be directed to
Messrs. Snowden & Cowman. Articles for publication should be received by
the editor at least three weeks previous to the first of the month on which the
number in which it is designed for them to appear, is issued.
fC^jT'The American Journal of Dental Science will contain fifty pages
of reading matter in each number, making a volume of about
Si* Hundred pages,
EXCLUSIVE OF ADVERTISEMENTS.
THifi
AMERICAN JOURNAL
OF
DENTAL SCIENCE.
The Journal is issued on the first of each month and contains not less
than fifty pages of reading matter in each number, exclusive of adver-
tisements, making a volume of six hundred pages per annum.
In each number "will be found Original Communications, Corres-
pondence, Selected Articles, Monthly Summary, Bibliographical No-
tices and Editorial Matter, and every effort will be exerted to render
the Journal worthy of the patronage of the Dental profession.
A generous co-operation is respectfully solicited from the members
s of the profession; and as the Journalhas now a printing office of its
own, which materially lessens the cost of its publication, it will be
issued at the reduced subscription price of
$2,SO Per Annum in jaLdvaxioe,
/ ?
Communications are respectfully solicited on all subjects pertain-
ing to the practice of dentistry, as well as reports of cases occurring
,in dental practice.
HATES OF ADVERTISING.
I Page.
1 MO.
$15 00
i " | 10 00
i !" I 7 00
J " I 5 00
2 MO.
$22 50
15 00
11 00
8 00
3 MO.
$30 00
20 00
15 00
11 00
6 MO. I 12 MO.
$50 00
80 00
20 00
15 00
$100 00
50 00
30 00
20 00
All original communications designed for publication, works for
review, new instruments for notice, exchanges, &c., should be directed
+ o the editor, 259 N. Eutaw St., Baltimore, Md.
All advertisements and subscriptions should be addressed to
SNOWDEN & COWMAN,
Publishers of the Am. Journal of Dental Science.
86 W. Fayette St. Bet. Charles & Liberty Sts.,
BALTIMORE. MD.

				

